# Differential Susceptibility to Benzo[a]pyrene Exposure during Gestation and Lactation in Mice with Genetic Variations in the Aryl Hydrocarbon Receptor and *Cyp1* Genes

**DOI:** 10.3390/toxics11090778

**Published:** 2023-09-13

**Authors:** Mackenzie Feltner, Patrick M. Hare, Asia Good, Emma G. Foster, Katelyn Clough, Jade Perry, Amanda Honaker, Angela Kyntchev, Mickayla Kowalski, Christine Perdan Curran

**Affiliations:** 1 Department of Biological Sciences, Northern Kentucky University, Highland Heights, KY 41099, USA; feltnerm2@nku.edu (M.F.); emmfoster@unmc.edu (E.G.F.); cloughk@unc.edu (K.C.); honakera2@nku.edu (A.H.); kyntcheva1@nku.edu (A.K.); kowalskim2@nku.edu (M.K.); 2 Department of Chemistry & Biochemistry, Northern Kentucky University, Highland Heights, KY 41099, USA; harep1@nku.edu

**Keywords:** polycyclic aromatic hydrocarbons, neonatal lethality, benzo[a]pyrene, aryl hydrocarbon receptor, CYP1A1, CYP1A2, CYP1B1

## Abstract

Polycyclic aromatic hydrocarbons are ubiquitous air pollutants, with additional widespread exposure in the diet. PAH exposure has been linked to adverse birth outcomes and long-term neurological consequences. To understand genetic differences that could affect susceptibility following developmental exposure to polycyclic aromatic hydrocarbons, we exposed mice with variations in the aryl hydrocarbon receptor and the three CYP1 enzymes from gestational day 10 (G10) to weaning at postnatal day 25 (P25). We found unexpectedly high neonatal lethality in high-affinity *Ahr^b^Cyp1b1(-/-)* knockout mice compared with all other genotypes. Over 60% of BaP-exposed pups died within their first 5 days of life. There was a significant effect of BaP on growth rates in surviving pups, with lower weights observed from P7 to P21. Again, *Ahr^b^Cyp1b1(-/-)* knockout mice were the most susceptible to growth retardation. Independent of treatment, this line of mice also had impaired development of the surface righting reflex. We used high-resolution mass spectrometry to measure BaP and metabolites in tissues from both dams and pups. We found the highest BaP levels in adipose from poor-affinity *Ahr^d^Cyp1a2(-/-)* dams and identified three major BaP metabolites (BaP-7-OH, BaP-9-OH, and BaP-4,5-diol), but our measurements were limited to a single time point. Future work is needed to understand BaP pharmacokinetics in the contexts of gestation and lactation and how differential metabolism leads to adverse developmental outcomes.

## 1. Introduction

Polycyclic aromatic hydrocarbons (PAHs) are complex organic molecules formed during combustion processes, the ubiquity of which results in widespread human exposure. The most common sources of exposure are traffic-related air pollution, fossil fuel burning, cigarette and wildfire smoke, and ingestion of grilled foods [1]. One of the most widely studied PAHs is the carcinogen benzo[a]pyrene (BaP), which is ranked eighth on the U.S. government’s list of priority pollutants [2]. Exposure varies widely depending on location; however, they have been reported to range from nearly 10,000 to over 42,000 ng BaP eq/d in areas near petrochemical facilities [3]. Interestingly, in North America and Europe, indoor air and dust represent significant sources of exposure, whereas indoor and outdoor exposures are similar in Asia [4]. Climate change is likely to increase exposure to wildfire smoke worldwide. The U.S. EPA reports an average of 70,000 wildfires per year, with more severe damage and more acres burned in recent years than previously recorded [5].

The biotransformation of ingested or inhaled PAHs results in reactive metabolites that can form DNA adducts, increasing the risk of mutations leading to cancer or birth defects [6,7]. Other adverse human health outcomes associated with PAH exposure include stunted growth in exposed children [8], metabolic syndrome [9], immune suppression [10,11], hypertension in both adults [12] and children [13], and cardiovascular disease [14]. Prospective human studies following pregnant women and their children over time recently demonstrated that prenatal and early-life PAH exposure are associated with numerous adverse outcomes, including cognitive impairments and behavioral problems that persist into adolescence [15,16,17,18,19].

The metabolism of PAHs is mediated by the aryl hydrocarbon receptor (AHR) and the CYP1 family of mono-oxygenase enzymes regulated by the AHR [20,21]. Activation of this pathway results in the production of toxic metabolites, increased oxidative stress, and potential excretion following conjugation reactions (reviewed in [22]). Data from the United States National Health and Nutrition Examination Survey (NHANES) demonstrated that PAH exposure varies across ethnic groups, with Hispanic and non-Hispanic Black populations at the highest risk [12]. Genetic differences in the AHR pathway are known to alter metabolism in both humans [23] and in animal models [24,25]. As a result, there can be significant differences in the pharmacokinetics of BaP and the internal dose, depending on an individual’s genotype. In adult mice, the lack of CYP1A1 led to higher levels after oral BaP exposure, while the presence of CYP1B1 was essential for immunotoxicity [24]. Our current work extends these studies by looking at genetic differences in the context of BaP exposure during gestation and lactation.

Our previous work, involving mice with allelic variations at the *Ahr* and *Cyp1a2* loci, confirmed that offspring exposed to BaP during gestation and lactation had differential susceptibility to early-life PAH exposure [26]. We found that both high-affinity *Ahr^b^Cyp1a2(-/-)* knockout mice and poor-affinity *Ahr^d^Cyp1a2(-/-)* mice had greater cognitive deficits and motor impairments as adults compared with controls and wild type mice. In our follow-up experiments, we expanded our studies to include all *Cyp1* knockout mice to determine the relative importance of each enzyme (CYP1A1, CYP1A2, and CYP1B1) in the metabolism and excretion of BaP in both dams and their offspring and how differences affected early development in the exposed offspring. Although we hypothesized that *Ahr^b^Cyp1a1(-/-)* mice would be vulnerable, based on previous studies [24,25,27], we were surprised to find that *Ahr^b^Cyp1b1(-/-)* mice were uniquely susceptible to developmental BaP toxicity.

## 2. Materials and Methods

### 2.1. Animals

Animal experiments were conducted under protocols approved by the Northern Kentucky University Institutional Animal Care and Use Committee (IACUC) and in line with the ARRIVE Guidelines [28,29]. Requirements for housing and care of animals followed the Guide for the Care and Use of Laboratory Animals (8th edition).

#### 2.1.1. Animal Sourcing and Husbandry

Male and female C57BL/6J mice were purchased from The Jackson Laboratory (Bar Harbor, ME). The following knockout mice were back-crossed onto a B6 background for at least eight generations: *Ahr^b^Cyp1a1(-/-)*, *Ahr^b^Cyp1a2(-/-)*, *Ahr^d^Cyp1a2(-/-)*, and *Ahr^b^Cyp1b1(-/-)*. Generation of the *Ahr^b^Cyp1a1(-/-)* mouse line has been described previously [30]. High-affinity *Ahr^b^Cyp1a2(-/-)* mice were generated by the Nebert Laboratory [31], and poor-affinity *Ahr^d^Cyp1a2(-/-)* mice were generated at the University of Cincinnati, as described in a previous study [32]. The generation of the *Ahr^b^Cyp1b1(-/-)* line is described in [33]. The colonies were maintained in the Northern Kentucky University vivarium. To avoid confounding through genetic drift, the knockout lines were routinely back-crossed to B6 breeders purchased from The Jackson Laboratory. Up to 4 animals of the same sex and genotype were group-housed in standard polysulfone shoebox cages with static microfilter lids, corncob bedding, and one cotton nestlet for enrichment. Housing rooms were set to a 12 h:12 h light–dark cycle. Water and Lab Diet 5015 chow (18.9% protein; 11% fat; 32.6% starch) were provided ad libitum. Cages were changed weekly, and daily animal checks were conducted to identify any health concerns. All animals were given >1 wk. to acclimate to the vivarium preceding any experiments. Both male and female offspring were used, to determine the effect of sex as a biological variable.

For behavioral studies, we used 10–15 litters per group. For body and tissue weights, we collected data from each dam and one male and one female pup per litter, with 10–15 litters per group. For BaP and metabolite measurements, we used 4–6 animals per group.

#### 2.1.2. Breeding

Nulliparous females were mated with males of the same genotype over a four-day breeding cycle. Dams were removed from the breeding cage once a vaginal plug was found. Litters were balanced to six pups per dam through culling or cross-fostering.

#### 2.1.3. Benzo[a]pyrene Treatment

Benzo[a]pyrene (BaP; >96% purity) was purchased from Millipore-Sigma (Burlington, MA, USA) and dissolved in corn oil. Pregnant dams were randomly assigned to treatment groups at gestational day 10 (G10): 10 mg/kg/d BaP in corn oil or 20 μL/d of the corn oil vehicle. To avoid the stress of gavage treatment, dosing solutions were pipetted onto small pieces of cereal (Cap’n Crunch™ peanut butter) and readily consumed by the dams. Treatments continued daily until pups were weaned at postnatal day 25 (P25). The dose of BaP was consistent with our previous studies to avoid overt toxicity [26], and to mimic known human exposures given the higher metabolic rate of laboratory mice [34]. The dosing period was chosen to cover the key neurodevelopmental periods seen in the second and third trimesters of human pregnancy [35].

#### 2.1.4. Verification of Genotype

All animals’ genotypes were confirmed with PCR genotyping of tail snips (~1 cm) at weaning and again after sacrifice.

### 2.2. Dam Behavior

Snapshot ethograms were used to assess dam behavior during the early postnatal period (P4–P6). The time spent on nest was recorded and compared across all treatment groups. Nest quality was assessed using a scale of 0–3, with 0 being the absence of a nest and 3 being a completely rounded nest with a roof over the pups.

### 2.3. Litter Viability and Pup Development

Litters were monitored for their total number of live pups and neonatal lethality. Pups were weighed weekly until weaning to assess growth. Standard developmental landmarks, such as pinna detachment, fur development, and eye opening, were monitored for the first two weeks of life.

### 2.4. Neonatal Reflexes

Two tests of neonatal reflexes were conducted to determine if there were developmental delays in any of the treatment groups, according to the methods of Feather-Schussler and Ferguson [36].

#### 2.4.1. Surface Righting Reflex

At P5, P7, and P10, pups were placed on their backs in a clean, polysulfone shoebox cage and timed until they turned onto all four paws. Pups not completing the task within the allotted 30 s time limit were assigned a score of 30 for that trial. Each pup received 3 trials at each time point, with a 60 s inter-trial interval.

#### 2.4.2. Negative Geotaxis

At P7, P10 and P14, pups were placed facing downward on a slanted board (45° angle) covered with a textured cloth to prevent slipping, and timed until they turned 180°. Pups not completing the task within the allotted 60 s limit were assigned a score of 60 for that trial. Each pup received 3 trials at each time point, with a 120 s inter-trial interval.

### 2.5. Tissue Collection

Tissues were collected from dams and their offspring once litters reached P25. Each collection was conducted between 3:00 pm and 6:00 pm to account for circadian rhythms. Organ weights were normalized to body weights. Sacrifices were performed via rapid decapitation. Collected tissues included: liver, abdominal fat, heart, ileum, duodenum, and trunk blood. Because a primary concern of the overarching study was BaP-induced neurotoxicity, cortex and cerebellum were also collected from the pups. All tissues were flash frozen and stored at −80 °C. When possible, tissues were collected from one male and one female per litter. Wet weights were recorded for liver, heart, thymus, and spleen after rinsing with phosphate-buffered saline and blotting.

### 2.6. Benzo[a]pyrene Extraction

Benzo[a]pyrene levels were measured in the liver and adipose of dams and pups when the pups reached P25. Levels of BaP were also analyzed in extracted pup cortex and cerebellum. Extraction methods were adapted from Uno et al. [25]. About 50 mg of each tissue was weighed. For liver and adipose, 1 mL of 2:1 *v*/*v* ethyl acetate/acetone was added in a glass culture tube and homogenized with a polytron homogenizer. For the brain tissues, 200 μL of USP saline and 1 mL of 2:1 *v*/*v* ethyl acetate/acetone were added and the tissue homogenized in a glass Dounce homogenizer. Samples were vortexed for 2 min using a Disruptor Genie^®^ (Scientific Industries, Bohemia, NY, USA), then centrifuged at 12,000 rcf for 3 min. The organic phase was collected. All tissues were extracted twice more with 100 μL 2:1 *v*/*v* ethyl acetate/acetone. The supernatants were evaporated under a gentle stream of ultra-high-purity argon gas at 40 °C. All samples were reconstituted in 200 μL acetonitrile and briefly vortexed. Each sample was filtered into a glass total recovery vial using a 1 mL syringe attached to a 0.45 μm 13 mm wwPTFE acrodisc filter (Cytiva Life Sciences, Marlborough, MA, USA).

### 2.7. Benzo[a]pyrene Quantification

Benzo[a]pyrene was detected and quantified using a Waters Xevo G2-X2 MS system with a Waters ACQUITY UPLC (Milford, MA). Samples were injected with the system in positive ion mode onto a Waters UPLC BEH C18 column (1.7 μm, 2.1 × 50 mm) with a Waters UPLC BEH C18 VanGuard Pre-Column (1.7 μm, 2.1 × 5 mm), at an injection volume of 0.5 μL. Mobile phase A was LC-MS grade acetonitrile, while mobile phase B was 0.1% formic acid in water from a milliQ purification system (Millipore-Sigma). The following solvent gradient was developed from [37] 35/65 (A/B, *v*/*v*) to 60/40 (A/B, *v*/*v*) from minutes 0–6, 60/40 (A/B, *v*/*v*) to 100/0 (A/B, *v*/*v*) from minutes 6 to 10, 35/65 (A/B, *v*/*v*) from minutes 10–11. The flow rate was 0.25 mL/min. Masses between 100 and 1500 Daltons were collected in centroid mode with a 0.1s scan time. The cone and capillary voltages were 80 V and 2.5 kV, respectively. The source temperature was 140 °C; the desolvation temperature was 600 °C. Mass calibration was performed daily with sodium formate. Mass accuracy was checked during sample runs with leucine enkephalin. Quantification was performed using Waters’ TargetLynx via integration of the 252.097 *m*/*z* channel, with a retention time window of 8.82 ± 0.1 min using a calibration with BaP. For the calibration, BaP standards were obtained by dissolving 10 mg BaP powder in 10 mL of LC-MS grade acetonitrile, creating a concentration of 1000 ng BaP/μL. Through serial dilutions, concentrations of 5 ng/μL, 2.5 ng/μL, 1 ng/μL, 0.5 ng/μL, and 0.1 ng/μL were prepared. BaP standards were injected onto the Waters ACQUITY UPLC system using the same method as described for the samples.

### 2.8. Benzo[a]pyrene Metabolite Quantification

Standards of: 3-, 7-, 9-, and 12-hydroxy-BaP; 4,5-, 7,8-, and 9,10- BaP-diols; and the 4,5-epoxide of BaP were obtained as 10 μM solutions in acetonitrile as a generous gift from Jamie M. Pennington, originally obtained as analytical standards from the Oregon State University Superfund Repository. They were injected onto the Waters AQUITY system under the same LC and MS conditions as those used for BaP measurements (Section 2.7). The hydroxy-BaP and epoxide compounds were monitored using the 269.09 *m*/*z* mass channel and appeared at retention times of 6.74, 6.77, 6.35, and 6.68 min for 3-, 7-, 9-, and 12-hydroxy-BaP, respectively. The epoxide was also found at a retention time of 6.68 min. The diols were monitored using the 287.099 *m*/*z* mass channel and appeared at retention times of 3.02, 3.21, and 1.97 min for the 4,5-, 7,8-, and 9,10- diols, respectively. Analysis was conducted using TargetLynx.

### 2.9. Data Analysis

Data were analyzed using SPSS statistical software (IBM Version 28.0) and SigmaPlot (Systat Software Version 13.0) to run General Linear Model ANOVAs examining the effects of genotype, treatment, and sex and to compare differences between dams and pups. To reduce animal numbers and because all mouse lines are from the same B6 genetic background, we compared BaP-treated mice to corn oil-treated C57BL/6J controls for multiple analyses using the Holm–Sidak method. When it was important to determine if genotype could be a critical factor, we included corn oil-treated animals from all genotypes. If differences or significant interactions were found, post hoc analyses using Bonferroni correction were conducted to identify which groups were different. Significance was set at 0.05. All data are presented as LS means, and error bars represent the standard error of the mean.

## 3. Results

### 3.1. BaP Effects on Dams

There was minimal effect of BaP treatment during gestation and lactation on the dams, regardless of genotype.

#### 3.1.1. Dam Weights

**Figure 1:**
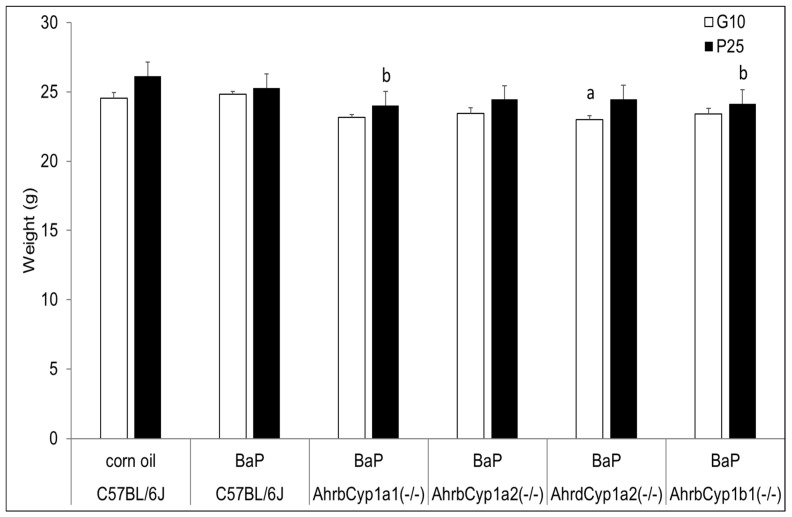
Dam weights at the beginning and end of BaP treatment. There was a significant effect of genotype at baseline (G10) for poor-affinity *Ahr^d^Cyp1a2(-/-)* dams and at weaning for high-affinity *Ahr^b^Cyp1a1(-/-)* and *Ahr^b^Cyp1b1(-/-)* mice. N = 10–15 litters per group; a = significantly different from C57BL/6J controls; b = significantly different from C57BL/6J; *p* < 0.05.

Although *Ahr^d^Cyp1a2(-/-)* knockout dams weighed significantly less than all other groups when treatment began at G10, their weights did not deviate from the controls or other groups at the end of treatment, when the pups were at P25. In contrast, both *Ahr^b^Cyp1a1(-/-)* and *Ahr^b^Cyp1b1(-/-)* knockout dams weighed significantly less at the end of the treatment period (Figure 1).

#### 3.1.2. Dam Behavior

There was no adverse effect of BaP treatment on dam behavior. All dams spent equivalent times on nest (*p* > 0.05), and all genotypes had nest quality scores equal to or exceeding those of corn oil-treated C57BL/6J control dams.

### 3.2. BaP Effects on Offspring

#### 3.2.1. Litter Viability

**Figure 2:**
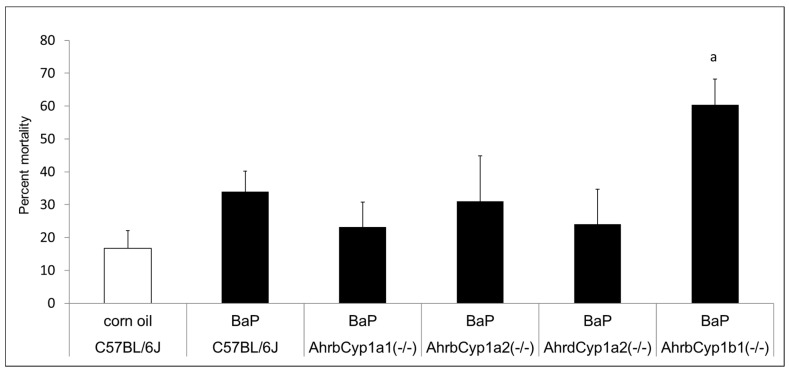
Neonatal lethality in BaP-treated wild type and knockout lines. Gestational and lactational treatment with BaP resulted in high litter losses for the *Ahr^b^Cyp1b1(-/-)* line. N = 10–15 litters per group; a = significantly different from C57BL/6J controls; *p* < 0.01.

There was no difference in the mean litter size. All genotypes and treatment groups averaged ~ seven pups per litter, which is expected from the C57BL/6J strain (The Jackson Laboratory). However, we discovered significant and strikingly high neonatal mortality in BaP-treated *Ahr^b^Cyp1b1(-/-)* offspring (Figure 2). Over 60% of pups born to these dams died within the first 5 days of life. Neonatal mortality was higher than control values for other groups, but those differences did not reach statistical significance (*p* > 0.05). In a follow-up experiment, we decreased the dose by 50% for *Ahr^b^Cyp1b1(-/-),* to 5 mg/kg/day, and still found high neonatal mortality, with 49% of the pups dying within the first 5 days of life. For surviving pups, there were no delays in the classic landmarks of pinna detachment, fur development, or eye opening.

#### 3.2.2. Pup Weights

**Figure 3:**
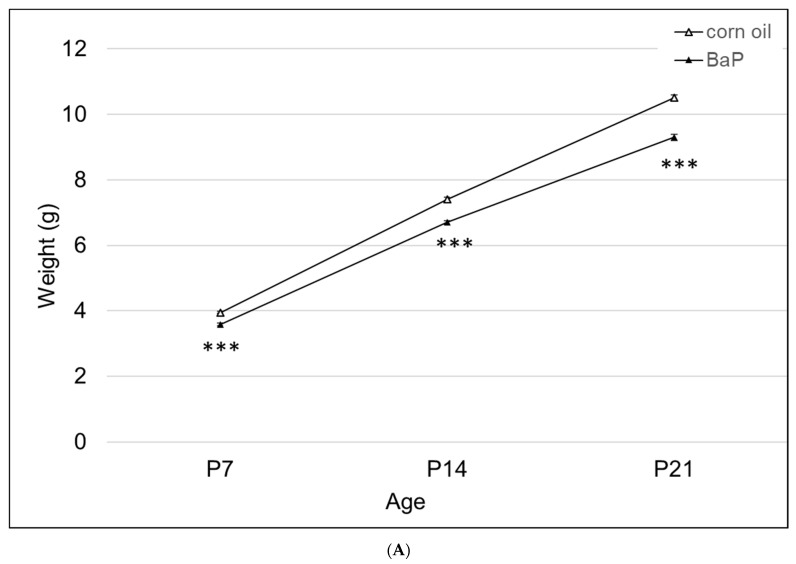

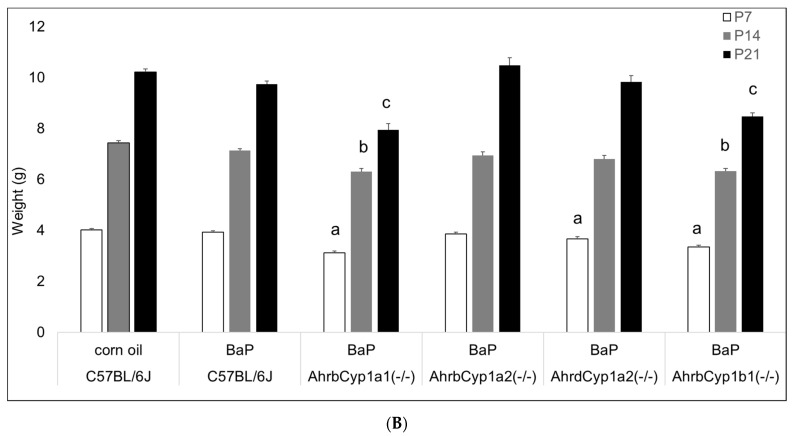
(**A**) Weight gain in offspring of the control and BaP-exposed offspring. There was a significant main effect of BaP treatment on weight in pups and a slightly slower growth rate in BaP treatment groups. All genotypes are pooled into one treatment group. N = 10–15 litters per genotype; *** *p* < 0.001. (**B**) Susceptible genotypes: there was a significant gene x treatment interaction for weight gain. *Ahr^b^Cyp1a1(-/-),* poor-affinity *Ahr^d^Cyp1a2(-/-)* and *Ahr^b^Cyp1b1(-/-)* knockout mice all weighed less than the controls at P7. At P14 and P21, the effects persisted in the *Ahr^b^Cyp1a1(-/-)* and *Ahr^b^Cyp1b1(-/-)* knockout mice. N = 10–15 litters per group; a = significantly different from controls at P7; b = significantly different from controls at P14; c = significantly different from controls at P21; *p* < 0.001 for all time points.

There was a significant effect of BaP exposure on pup growth, with lower weights at all time points tested (Figure 3A). Three of the four knockout lines had lower weights at P7, with *Ahr^b^Cyp1a1(-/-)* and *Ahr^b^Cyp1b1(-/-)* knockouts weighing significantly less than the controls throughout the postnatal period (Figure 3B).

#### 3.2.3. Thymus and Spleen Weights

**Figure 4:**
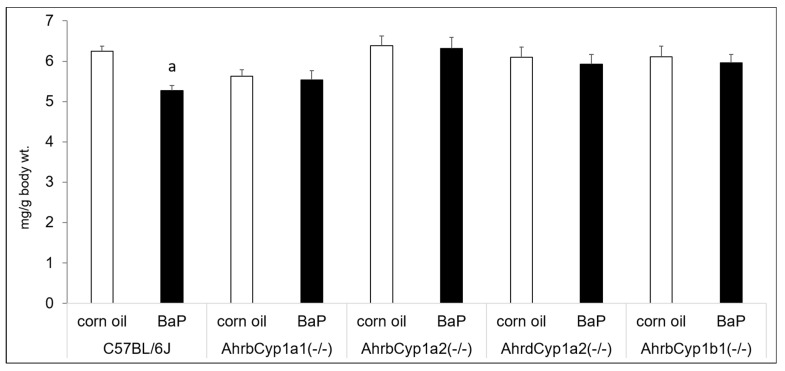
Spleen weights at P25. There was a significant gene x treatment interaction, with BaP-exposed C57BL/6J offspring having lower spleen weights, whereas all other genotypes showed no evidence of immune suppression. N = 10–15 litters per group; a = significantly different from corn oil-treated controls; *p* < 0.05.

Benzo[a]pyrene and other PAHs have been linked with immune suppression, but we did not find any effects of BaP exposure on thymus wet weights after controlling for body size. We did find a gene x treatment interaction for spleen weights. Interestingly, only wild type C57BL/6J offspring had reduced spleen weights in BaP-exposed animals compared with corn oil-treated controls (Figure 4).

#### 3.2.4. Surface Righting Reflex

**Figure 5:**
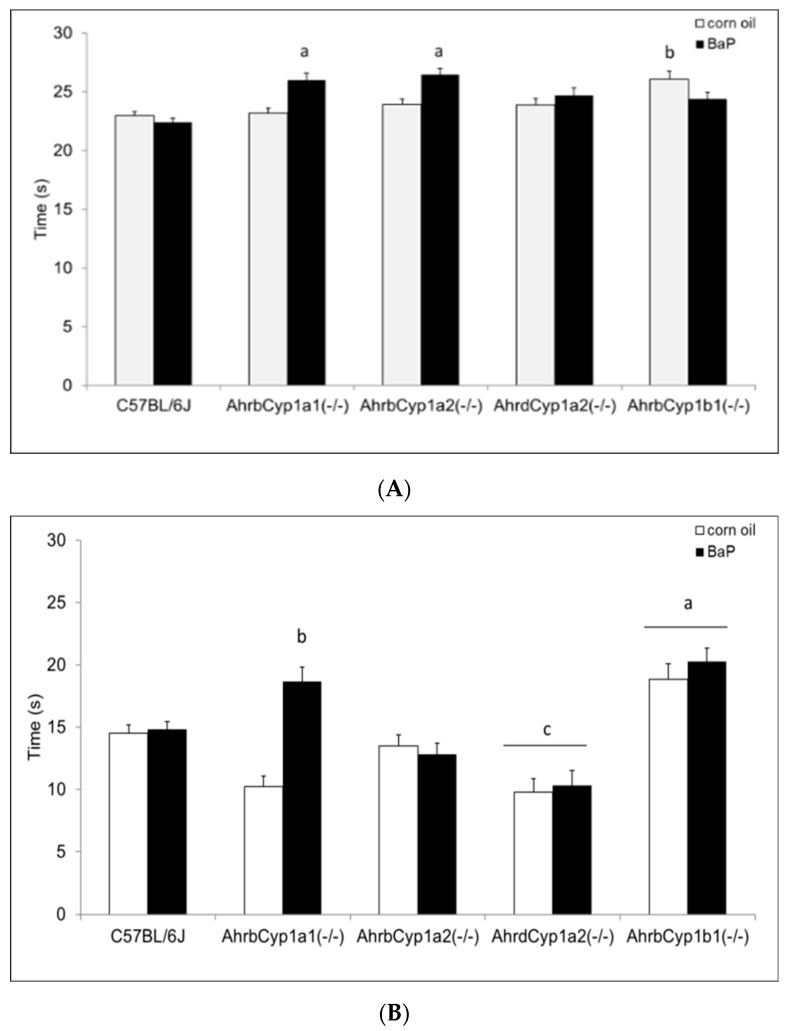

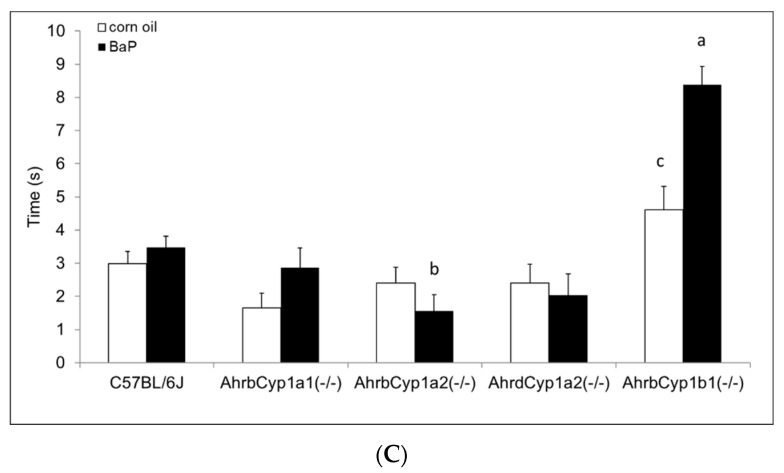
(**A**) Surface righting reflex at P5: impairments were seen in BaP-exposed high-affinity *Ahr^b^Cyp1a1(-/-), Ahr^b^Cyp1a2(-/-),* and corn-oil treated *Ahr^b^Cyp1b1(-/-)* mice. N = 10–15 litters per group; a = significantly slower than BaP-treated C57BL/6J mice, *p* < 0.05; b = significantly slower than corn oil-treated C57BL/6J, *p* < 0.01. (**B**) Surface righting reflex at P7: impairments were seen in BaP-exposed high-affinity *Ahr^b^Cyp1a1(-/-)* and all *Ahr^b^Cyp1b1(-/-)* mice. Both groups of *Ahr^d^Cyp1a2(-/-)* mice were faster than the controls. N = 10–15 litters per group; a = significantly slower than all other genotypes, *p* < 0.001; b = significantly slower than BaP-treated C57BL/6J, *p* < 0.001; c = significantly faster than BaP-treated C57BL/6J, *p* < 0.001. (**C**) Surface righting reflex at P10: impairments were seen in BaP-exposed high-affinity *Ahr^b^Cyp1a1(-/-)* and corn-oil treated *Ahr^b^Cyp1b1(-/-)* mice. In contrast, BaP-exposed *Ahr^b^Cyp1a2(-/-)* mice outperformed controls. N = 10–15 litters per group; a = significantly slower than all other genotypes, *p* < 0.001; b = significantly faster than BaP-treated C57BL/6J, *p* < 0.001; c = significantly slower than corn oil-treated *Ahr^b^Cyp1a1(-/-)*, *p* < 0.01.

There was a significant gene x treatment interaction for the surface righting reflex test. At P5, both *Ahr^b^Cyp1a1(-/-)* and *Ahr^b^Cyp1a2(-/-)* mice completed the task more slowly than the controls. In contrast, corn oil-treated *Ahr^b^Cyp1b1(-/-)* mice took longer than wild type mice, but BaP-exposed *Ahr^b^Cyp1b1(-/-)* mice were unaffected (Figure 5A). Greater differences were seen at P7, with BaP-exposed *Ahr^b^Cyp1a1(-/-)* and all *Ahr^b^Cyp1b1(-/-)* mice showing significant impairments, whereas treated and control *Ahr^d^Cyp1a2(-/-)* mice completed the task significantly faster than the controls (Figure 5B). BaP-exposed *Ahr^b^Cyp1b1(-/-)* mice were significantly impaired compared to all other genotypes at P10. Corn oil-treated *Ahr^b^Cyp1b1(-/-)* mice were significantly slower than the C57BL/6J controls, and BaP-exposed *Ahr^b^Cyp1a2(-/-)* mice were faster than the controls when they reached P10 (Figure 5C).

#### 3.2.5. Negative Geotaxis

**Figure 6:**
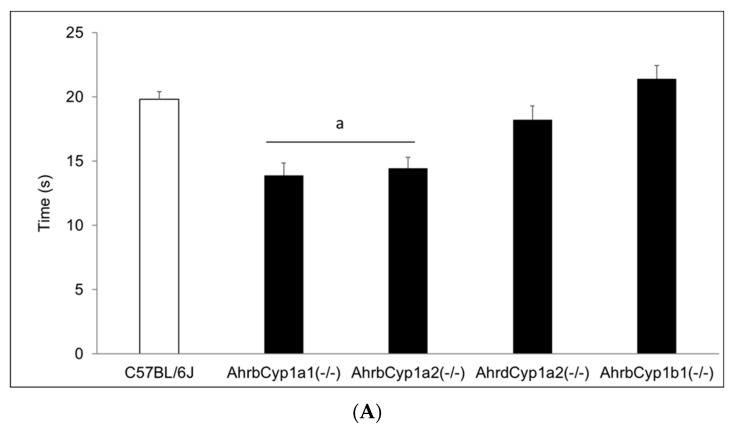

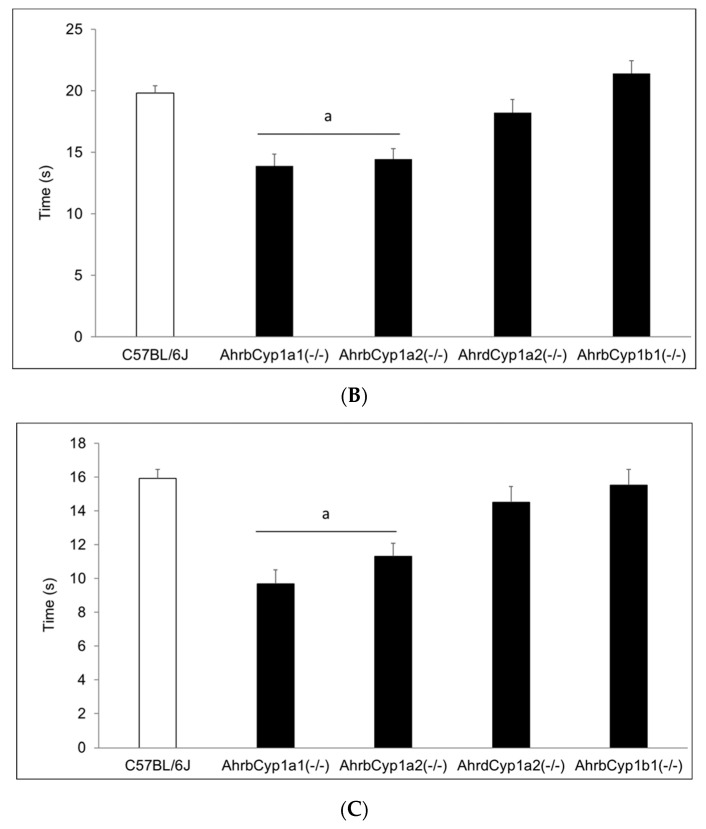
(**A**) Negative geotaxis at P7: high-affinity *Ahr^b^Cyp1a1(-/-)* and *Ahr^b^Cyp1a2(-/-)* knockouts had the shortest latencies to complete the 180° turn. N = 10–15 litters per group; a = significantly different from all other genotypes, *p* < 0.001. (**B**) Negative geotaxis at P10: high-affinity *Ahr^b^Cyp1a1(-/-)* and *Ahr^b^Cyp1a2(-/-)* knockouts had the shortest latencies to complete the 180° turn. N = 10–15 litters per group; a = significantly different from all other genotypes, *p* < 0.001. (**C**) Negative geotaxis at P14: high-affinity *Ahr^b^Cyp1a1(-/-)* and *Ahr^b^Cyp1a2(-/-)* knockouts had the shortest latencies to complete the 180° turn. N = 10–15 litters per group; a = significantly different from all other genotypes, *p* < 0.001.

There was no effect of treatment and no gene x treatment interaction for the negative geotaxis results on any of the three days of testing; however, there was a significant main effect of genotype (Figure 6A–C). *Ahr^b^Cyp1a1(-/-)* and *Ahr^b^Cyp1a2(-/-)* knockout mice performed the task faster than all other genotypes. No impairments were found.

### 3.3. Benzo[a]pyrene Distribution

**Figure 7:**
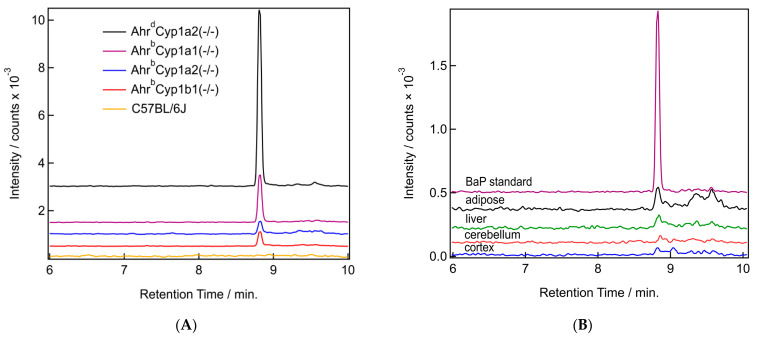
(**A**,**B**) Sample chromatograms for benzo[a]pyrene. (**A**) Chromatograms from the BaP mass channel (252.097 *m*/*z*) for all studied genotypes. All samples are from adipose tissue. (**B**) Chromatograms from the BaP mass channel (252.097 *m*/*z*) from a BaP in acetonitrile standard (purple) and samples from all four tissue types (adipose in black, liver in green, cerebellum in red, and cortex in blue) from the same *Ahr^d^Cyp1a2(-/-)* pup.

We were able to detect the parent compound benzo[a]pyrene in all tissues tested, with clear differences based on tissue type and genotype. BaP levels were the highest in the adipose of dams and in *Ahr^d^Cyp1a2(-/-)* mice compared with all other genotypes. Sample chromatograms demonstrate that levels were often undetectable in wild type C57BL/6J mice which have all three CYP1 enzymes present (Figure 7A). Levels were also lower in the cortex compared with the adipose, liver, and cerebellum (Figure 7B).

#### 3.3.1. BaP Levels in Adipose and Liver

The distribution of BaP in tissues had a consistent pattern when comparing dams with their pups. BaP levels were higher in the dams compared with their pups. This was expected, because all dosing was provided to the dam, and BaP was transferred to pups in utero or through lactation. The concentration found in adipose was higher compared with the concentration found in liver tissue. When adipose was compared, the highest levels of BaP were found in poor-affinity *Ahr^d^Cyp1a2(-/-)* knockout mice, followed by *Ahr^b^Cyp1a1(-/-)* mice. Notably, *Ahr^b^Cyp1a1(-/-)* pups had nearly the same concentration of BaP in adipose as their dams. This was the only genotype and tissue where this pattern was observed. The highest BaP levels in liver tissues were found in *Ahr^d^Cyp1a2(-/-)* mice (Table 1).

#### 3.3.2. BaP Levels in Brain

No significant differences were found when comparing BaP levels in the brains of P25 pups (Table 2). Levels were extremely low compared with levels found in adipose and liver tissues, and BaP was undetectable in the cortex of *Ahr^b^Cyp1b1(-/-)* knockout pups, as well as in the cerebellum of wild type C57BL/6J pups. Conversely, the highest levels of BaP were found in the cerebellum tissues of the *Ahr^b^Cyp1b1(-/-)* pups. We did not measure BaP levels in the dam brains, since our behavioral assessment of maternal care did not reveal any obvious neurological impairments.

### 3.4. BaP Metabolism

**Figure 8:**
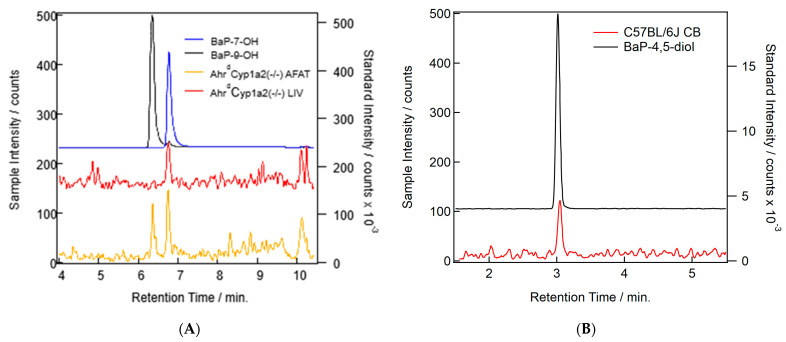
(**A**) Example chromatograms showing the probable detection of BaP metabolites in *Ahr^d^Cyp1a2(-/-)* liver (red) and adipose (orange) samples. Standards for BaP-7-OH (blue) and BaP-9-OH (black) are shown. (**B**) Example chromatograms showing the probable detection of the BaP-4,5-diol metabolite in a C57B/6J cerebellum (CB) sample (red) and the standards for the metabolite (black).

Our methods were able to separate BaP and all major metabolite standards. Only three of the common well-known BaP metabolites were found in our samples: BaP-7-OH, BaP-9-OH, and BaP-4,5-diol (Table 3). Concentrations were too low to quantify and compare across all groups, so no analysis was conducted. In addition to the peaks found (Figure 8A,B), we also found some other putative metabolite peaks that did not match the retention times for any of our standards. Since conjugation is a common Phase II reaction in BaP metabolism [22], further work will be needed to determine the identity of those molecules and their relevance to developmental BaP exposure.

## 4. Discussion

### 4.1. Unexpectedly High Neonatal Lethality Associated with Lack of CYP1B1

A major finding of this study was the significantly and surprisingly high neonatal lethality in offspring born to *Ahr^b^Cyp1b1(-/-)* knockout dams exposed to 10 mg/kg/d oral BaP starting at G10. All litter losses occurred within the first 5 days after birth. We found ~60% of the offspring did not survive, whereas all other BaP-exposed genotypes had litter losses comparable to the corn oil-treated C57BL/6J controls (Figure 2). Our analysis of dam behavior found no difference in maternal care; therefore, it is unlikely that maternal neglect was a factor. The dam behavior findings were also consistent with our previous studies of *Cyp1* knockout mice treated with AHR agonists [26,32]. We also found no significant differences in litter size, indicating that lethality was limited to the postnatal period. The fact that many *Ahr^b^Cyp1b1(-/-)* knockout dams raised successful litters to weaning at P25 also argues against lactational failure, although we acknowledge that growth rates were significantly lower in this line of mice (Figure 3A,B).

The observed neonatal lethality was similar to findings in BaP-exposed zebrafish larvae that suffered high mortality between 3 and 5 dpf [38]. In addition, Ye et al. [39] reported a dose-dependent increase in fetal resorptions and abnormalities in C57BL/6 mice treated with 0.05 to 2 mg/kg/d BaP from G1–10. The importance of BaP metabolism in lethality was highlighted in a recent study that found women who suffered fetal losses during early pregnancy (≤12 wk) had higher BPDE-DNA adduct levels compared to women with normal pregnancies. Moreover, the researchers found a clear dose-dependent response, with the highest adduct levels in chorionic villus tissue associated with the highest risk of fetal loss [40].

These studies, together with our previous work, implicate activation of the AHR pathway as a key initiating event in developmental toxicity. We found that a single i.p. injection of the AHR agonist, coplanar hexabromobiphenyl, at G5 resulted in a dose-dependent increase in litter losses for C57BL/6J mice with a high-affinity *Ahr* genotype [41]. We also found a significantly higher rate of dystocia and abnormal gestation lengths in two knockouts lines treated with a mixture of coplanar and non-coplanar polychlorinated biphenyls. Both high-affinity *Ahr^b^Cyp1a2(-/-)* and poor-affinity *Ahr^d^Cyp1a2(-/-)* knockouts were affected [42,43]. A key difference is that BaP is readily metabolized following AHR activation and CYP1 upregulation, while higher molecular weight polybrominated and polychlorinated biphenyls (PBBs and PCBs) are persistent organic pollutants that tend to bioaccumulate and resist degradation [44,45]. The mechanism of offspring toxicity for PBBs and PCBs has been clearly linked to a lack of maternal CYP1A2, which can sequester coplanar PBBs and PCBs, preventing their distribution to the fetus and pups [43,46]. The mechanism for BaP developmental toxicity is likely to be more complicated, given that more than one genotype had adverse effects.

### 4.2. Differential Metabolism of BaP in Mice and Humans

The difference in genotype susceptibility and the difference in pharmacokinetics for BaP led us to examine the tissue levels of BaP and its metabolites in both dams and pups. BaP exerts its toxicity partly through reactive metabolites [47], so determining which metabolites are present, and in what concentrations, is likely to provide a deeper understanding of the unique susceptibility of *Ahr^b^Cyp1b1(-/-)* knockout mice. We measured the BaP parent compound and tis metabolites in the liver and adipose of dams and pups, and in the cortex and cerebellum of P25 pups, using the littermates of animals bred for neurobehavioral studies. We had hypothesized that *Ahr^b^Cyp1a1(-/-)* knockout mice would have higher BaP levels, based on earlier pharmacokinetic studies of adult mice dosed with oral BaP [24,25,27]. We also anticipated that more BaP would be found in dams compared with pups, since all exposures occurred through treatment of the dams alone. Both of these hypotheses were supported by the data (Table 1); however, we found the highest BaP concentrations in the adipose tissue of poor-affinity *Ahr^d^Cyp1a2(-/-)* dams. This mirrors our findings with highly metabolizable PCB 77, which was found in significantly higher concentrations in the same genotype of mice [43]. In both cases, it is likely that failure to upregulate CYP1A1 was the primary factor limiting metabolism and excretion of the parent compounds.

In many cases, the levels of BaP were below the method’s limit of quantification. We were also unable to quantify the metabolites, although we did identify the presence of three well-known metabolites: BaP-7-OH, BaP-9-OH, and BaP-4,5-diol. The low levels of BaP and its metabolites were likely impacted by the experimental conditions. In addition to faster metabolism and excretion by wild type C57BL/6J, the tissues were collected ~24 h after the last dose was administered. This means that all animals with CYP1A1 and/or CYP1B1 present would have been able to undergo extensive metabolism and excretion prior to the time point when our measurements were made. We also did not measure blood levels of BaP during gestation and lactation, to avoid confounding our behavioral studies with maternal and offspring stressors. This is a limitation that will be addressed in our future studies.

Maier et al. [47] found significant variation in metabolite profiles and pharmacokinetics following oral dosing with ^14^C-labeled BaP. In contrast to our results, the predominant metabolites were tetrols, followed by diols. However, they also found metabolites that could not be identified with the standards available. In a follow-up study, the group showed that co-exposure to a second PAH, phenanthrene, reduced BaP absorption, but did not affect clearance or alter the metabolite profile [48].

Although the AHR-CYP1 pathway is directly activated by coplanar molecules, such as BaP and other PAHs, there are numerous other metabolic pathways involved that could affect both BAP toxicity and genetic susceptibility to developmental BaP exposure. These include enzymes involved in Phase II metabolism and conjugation pathways, such as glucuronosyltransferase, sulfotransferase, and glutathione-S-transferase. Because BaP metabolism by CYP enzymes leads to reactive intermediates and DNA adducts, polymorphisms in genes for the proteins involved in the oxidative stress response and DNA repair could also be associated with increased risk or resistance. For a detailed review, see Bukowska et al. [22].

### 4.3. Minimal Evidence of BaP-Induced Immunotoxicity

AHR agonists are often associated with immune suppression [49], which can be assessed by comparing thymus and spleen weights [50]. We found significantly decreased spleen weights only in BaP-exposed wild type C57BL/6J offspring at P25, and no change in thymus weights. These findings are similar to those of our previous work [43], in which the greatest difference at weaning was seen in wild type mice spleen weights, with no differences in thymus weight. For the doses used in this study, it does not appear that BaP had major immunotoxic effects. However, that observation must be tempered by recent findings that BaP alone does not accurately reflect the impact of more complex PAH mixtures on monocyte-to-macrophage differentiation [10]. Burchiel and Luster [11] also reported that low-dose PAH exposure could potentially enhance immune function by stimulating cell signaling pathways. This is biologically plausible, given that AHR activation can lead to both cell cycle progression and cell cycle arrest in both tissue- and context-specific manners [51]. Future studies could examine bone marrow and immune system challenges, which would provide more sensitive endpoints of immunotoxicity.

### 4.4. Strong Evidence of Genetic Susceptibility for Developmental Delays

There was a significant effect of BaP exposure on growth rates (Figure 3A), with the greatest effects on high-affinity *Ahr^b^Cyp1a1(-/-)* and *Ahr^b^Cyp1b1(-/-)* mice (Figure 3B). Depletion of thyroid hormones is often associated with growth retardation and is commonly reported following exposure to AHR agonists [52,53]. Recently, BaP was identified as an endocrine disrupter with effects on the development of the hypothalamus–pituitary–thyroid axis in larval zebrafish, although the mechanism of toxicity was not elucidated [38]. Therefore, future studies should measure levels of circulating thyroxine and the expression of thyroid hormone-dependent genes to determine if BaP has similar effects in mammalian systems. These studies would also help to determine if BaP is directly toxic to the thyroid gland or interferes with hormone transport or activation.

Neonatal reflexes developed differently, with a significant gene x treatment interaction only for the development of the surface righting reflex (Figure 5A–C). BaP-exposed high-affinity *Ahr^b^Cyp1a1(-/-)* mice were delayed at P5 and P7, but caught up to the controls by P10. High-affinity *Ahr^b^Cyp1a2(-/-)* mice outperformed controls at P7. Similarly, poor-affinity *Ahr^d^Cyp1a2(-/-)* mice outperformed controls at P10. *Ahr^b^Cyp1b1(-/-)* mice had impairments regardless of treatment, but BaP-exposed knockouts had the greatest delays across all three time points. This indicates that CYP1B1 metabolism could be more important than CYP1A1 during this window of brain development, while the absence of CYP1A2 had no adverse effects. This conclusion is strengthened by the negative geotaxis data (Figure 6A–C), in which high-affinity *Ahr^b^Cyp1a1(-/-)* and *Ahr^b^Cyp1a2(-/-)* knockouts outperformed controls. 

Studies in Sprague Dawley rats exposed to a PCB/organochlorine mixture also found delays in the surface righting reflex and negative geotaxis [54]. In contrast to our studies, the authors only found high mortality in offspring exposed throughout gestation and lactation. There were no adverse effects on neonatal lethality when the exposure window was limited to G11–17. It should be noted that the PCB mixture used in this study included both planar congeners that bind the AHR and non-coplanar congeners that activate other metabolic pathways. The mechanism of toxicity was not determined, but the authors did conclude that thyroid hormone disruption was not responsible. Studies in fish also found developmental neurotoxicity in larvae exposed to BaP. Knecht et al. [55] reported that zebrafish exposed to 4 µM BaP from 6 to 120 h post-fertilization were hyperactive, but larvae lacking AHR2 were not. Similarly, Brown et al. [56] reported that offspring of killifish raised in an unpolluted environment were susceptible to a PAH mixture and showed a similar hyperactive phenotype. Together, these findings further support our conclusion that AHR activation is a key initiating event in developmental BaP toxicity.

### 4.5. Relevance to Human Health

There is widespread exposure to polycyclic aromatic hydrocarbons from traffic-related air pollution, fossil fuel combustion, cigarette smoking, grilled foods, and wildfires [1]. Dietary intake was estimated at ~125 ng/kg/d for a typical Korean diet containing fried chicken [57], but the method of cooking greatly affects individual exposure. Smoked foods generally have the highest concentration, followed by grilled, roasted, and fried foods, raising the overall exposure 100-fold higher than in uncooked food [14]. Thai et al. [58] reported that urinary PAH levels showed an inverse relationship with economic status. Levels were lower in high-income countries and higher in low-to-middle-income countries. PAH levels in breast milk and umbilical cord blood are strong indicators of widespread exposure during pregnancy and lactation [18,59]. Therefore, PAHs remain a widespread hazard to human health.

Benzo[a]pyrene is a well-characterized PAH, metabolized by the CYP1 family of enzymes to reactive intermediates that can damage DNA and increase oxidative stress. The aryl hydrocarbon receptor is a ligand-activated transcription factor that upregulates all three CYP1 enzymes, but CYP1A1 is unique because it is inducible, and generally not found in any tissue until the AHR is activated [21]. CYP1A2 is present in the liver at a basal level and can be induced to higher levels, while CYP1B1 is normally present in endocrine tissues, but can be induced in most other tissues after AHR activation [60]. Polymorphisms in both the AHR and CYP1 family members are known, and many are linked to adverse health outcomes in humans [27,60]. This suggests that additional monitoring and genetic testing are warranted to identify the human populations at highest risk of developmental PAH exposure.

PAH exposure can have significant impacts on the developing fetus, including decreased head circumference, lower birth weight, lower body mass index, and lower APGAR scores [61]. Cognitive and behavioral effects persist into school age and adolescence [15,16]. PAH exposure has also been linked to an increase in congenital heart defects [62,63,64], and AHR agonists have been repeatedly shown to cause heart malformations in animal models [65,66]. Therefore, future work should also consider whether the susceptible *Ahr^b^Cyp1b1(-/-)* knockout mice had a higher rate of BaP-induced congenital heart defects. Since these are the most common birth defects, but most are of unknown etiology [67], this line of research could have the most important impacts on human health.

## 5. Conclusions

Using a mouse model with variations at the *Ahr* and *Cyp1* loci, we uncovered unexpected and unusually high neonatal lethality in high-affinity *Ahr^b^Cyp1b1(-/-)* mice when their dams were exposed to BaP during gestation and lactation. These mice also showed greatest impairments in growth rate and the development of neonatal reflexes. Measurements of BaP levels in four tissues indicate that BaP was transferred to offspring, and levels were well within the range of known human exposures. Three BaP metabolites were identified, but could not be quantified due to low levels. Genetic differences in our mouse model mimic known human genetic variation. These results indicate that further work is needed to better understand the pharmacokinetics of BaP and the role of the AHR-CYP1 pathway in the context of pregnancy and gestation.

## Figures and Tables

**Table 1:** There was a main effect of genotype for BaP levels in both adipose and liver. **Bold text** indicates groups with significantly higher levels than all other genotypes. N = 4–6 mice per group; *p* < 0.001.

Distribution of BaP in Dams and Pups			
Genotype		Adipose (ng/mg Tissue)	Liver (ng/mg Tissue)
C57BL/6J	dam	0.031 ± 0.14	0.001 ± 0.02
	pup	0.006 ± 0.13	0.017 ± 0.01
***Ahr^b^Cyp1a1(-/-)***	dam	**0.330 ± 0.22**	0.009 ± 0.01
	pup	**0.259 ± 0.16**	0.005 ± 0.01
*Ahr^b^Cyp1a2(-/-)*	dam	0.050 ± 0.16	0.003 ± 0.01
	pup	0.026 ± 0.13	0.004 ± 0.01
***Ahr^d^Cyp1a2(-/-)***	dam	**2.059 ± 0.15**	**0.074 **± 0.02
	pup	**0.031 ± 0.14**	**0.023** ± 0.02
*Ahr^b^Cyp1b1(-/-)*	dam	0.057 ± 0.12	0.012 ± 0.02
	pup	0.012 ± 0.11	0.031 ± 0.01

**Table 2:** There were no significant differences when comparing BaP levels in the cortex and cerebellum. N = 4–6 mice per group; *p* > 0.05.

Distribution of BaP in Pup Brain		
Genotype	Cortex (ng/mg Tissue)	Cerebellum (ng/mg Tissue)
C57BL/6J	0.006	ND
*Ahr^b^Cyp1a1(-/-)*	0.007	0.036
*Ahr^b^Cyp1a2(-/-)*	0.007	0.043
*Ahr^d^Cyp1a2(-/-)*	0.011	0.009
*Ahr^b^Cyp1b1(-/-)*	ND	0.085

**Table 3:** Retention times, in minutes, and monitored mass channels ([M+H]^+^) for BaP metabolite standards.

Identification of BaP Metabolites			
Metabolite	*m*/*z*	Retention Time	Found
BaP-3-OH	269.09	6.74	
BaP-7-OH	269.09	6.77	X
BaP-9-OH	269.09	6.35	X
BaP-12-OH	269.09	6.68	
BaP-4,5-diol	287.099	3.02	X
BaP-7,8-diol	287.099	3.21	
BaP-9,10-diol	287.099	1.97	
BaP-4,5-epoxide	269.09	6.68	

## Data Availability

All data and detailed protocols are freely available upon request to the corresponding author.
